# Multidimensional assessment of personality disorders using different theoretical models: a comparison of the Young Schema Questionnaire, the SCID-5-AMPD structured diagnostic interview, and the PDS-ICD-11 self-report questionnaire

**DOI:** 10.1192/j.eurpsy.2024.1392

**Published:** 2024-08-27

**Authors:** V. Pribula, J. Biliczki, L. Király, F. Pongracz, P. Ruscsak, N. B. Vadon, T. A. Renko, B. Erdelyi-Hamza, H. Szocs, G. Vizin, X. Gonda

**Affiliations:** ^1^ Institute of Psychology, Eotvos Lorand University; ^2^Department of Crisis Intervention and Psychiatry, Peterfy Sandor Street Hospital and Clinic; ^3^ Institute of Psychology, Eotvos Loránd University; ^4^Department of Psychiatry and Psychotherapy; ^5^Department of Clinical Psychology, Semmelweis University, Budapest, Hungary

## Abstract

**Introduction:**

There has been a recent shift in the conceptualisation of personality disorders in diagnostic systems such as DSM-5 or ICD-11, from a categorical approach towards a dimensional approach reflecting severity in general or severity of dysfunction and related pathological traits. In addition, several psychotherapeutic approaches work with their own model of personality pathology, which similarly capture symptoms of personality disorders and their underlying processes in a more subtle way from multiple aspects, and along different constructs.

**Objectives:**

The aim of our study was to investigate similarities and differences between conceptualisations of personality disorder and instruments used for evaluation based on the BNO-11 Personality Disorders Severity Questionnaire (PDS-ICD-11), Module I. of the Structured Diagnostic Interview for the DSM-5 Alternative Personality Model (SCID-5-AMPD) measuring level of personality function, and the Young Schema Questionnaire assessing early maladaptive schemas.

**Methods:**

Hospitalized borderline patients were assessed using the Young Schema Questionnaire, the PDS-ICD-11, and Module I. of the SCID-5-AMPD assessing personality function level. Data are analysed using correlation and linear regression models.

**Results:**

Only part of the results are shown. The PDS-ICD-11 Severity Index and Self-function Index showed significant (p<0.05) and strong correlations with the Abandonment (r=0.98, r=0.94), Vulnerability to harm and illness (r=0. 92, r=0.98), Insufficient Self-Control (r=0.91, r=0.88) and Negativism/Pessimism (r=0.95, r=0.90) schemas. The mean score and all domains of the SCID-5-AMPD Module I (level of personality function) showed significant strong correlations with the Vulnerability to harm and illness schema (AMPD-Average r=0.87; AMPD-Identity r=0.86, AMPD-Objectivity r=0.81, AMPD-Empathy r=0. 77, AMPD-Intimacy r=0.80, p<0.05); moreover, a strong significant correlation was found between the Abandonment schema and AMPD-Average (r=0.81, p<0.05), AMPD-Identity (r=0.98, p<0.05), and AMPD-Intimacy domains (r=0.77, p<0.05).

**Conclusions:**

The main indicators of measures that operationalise a dimensional approach to personality disorders show distinct patterns of strong overlap with some of the maladaptive schemas but cover only a part of the schema domains. For a careful diagnosis and psychotherapeutic plan, the combined use of these measures can provide in-depth and multifaceted information.

**Disclosure of Interest:**

None Declared

